# Peptide Hormones in the Insect Midgut

**DOI:** 10.3389/fphys.2020.00191

**Published:** 2020-03-05

**Authors:** Kai Wu, Shirong Li, Jing Wang, Yuyang Ni, Wuren Huang, Qiuning Liu, Erjun Ling

**Affiliations:** ^1^College of Life Sciences, Shangrao Normal University, Shangrao, China; ^2^Key Laboratory of Insect Developmental and Evolutionary Biology, Chinese Academy of Sciences Center for Excellence in Molecular Plant Sciences, Shanghai Institute of Plant Physiology and Ecology, Chinese Academy of Sciences, Shanghai, China; ^3^Jiangsu Key Laboratory for Bioresources of Saline Soils, Jiangsu Synthetic Innovation Center for Coastal Bio-Agriculture, Jiangsu Provincial Key Laboratory of Coastal Wetland Bioresources and Environmental Protection, School of Wetland, Yancheng Teachers University, Yancheng, China; ^4^Innovative Academy of Seed Design, Chinese Academy of Sciences, Beijing, China

**Keywords:** insect, peptide hormones, midgut, symbionts, immunity, bio-control

## Abstract

Insects produce many peptide hormones that play important roles in regulating growth, development, immunity, homeostasis, stress, and other processes to maintain normal life. As part of the digestive system, the insect midgut is also affected by hormones secreted from the prothoracic gland, corpus allatum, and various neuronal cells; these hormones regulate the secretion and activity of insects’ digestive enzymes and change their feeding behaviors. In addition, the insect midgut produces certain hormones when it recognizes various components or pathogenic bacteria in ingested foods; concurrently, the hormones regulate other tissues and organs. In addition, intestinal symbiotic bacteria can produce hormones that influence insect signaling pathways to promote host growth and development; this interaction is the result of long-term evolution. In this review, the types, functions, and mechanisms of hormones working on the insect midgut, as well as hormones produced therein, are reviewed for future reference in biological pest control.

Insect hormones are secreted by the endocrine system within the body and participate in the regulation and control of processes such as growth, metamorphosis, and reproduction (Jindra et al., 2013; Slama, 2015; Roy et al., 2018; Santos et al., 2019). In contrast, pheromones, also known as chemical signals, that can allow insects to communicate within species (Yew and Chung, 2015). Insect hormones can be categorized into steroids, sesquiterpenes, polypeptides, and others. Ecdysone is a steroid hormone synthesized by the prothoracic gland (PG) (Ou et al., 2016). Juvenile hormone (JH) is a sesquiterpene compound produced and secreted by the corpus allatum (Roller et al., 1967; Jindra et al., 2013). These two hormones are very important for insect growth and development. In addition, numerous peptide hormones in insects also exhibit important regulatory effects on insect growth and development. Insect endocrine peptides can have three main functions: promotion of digestion, transmission of information to the central nervous system, and promotion of nutrient absorption and conversion (Wegener and Veenstra, 2015). Peptide hormones also affect the synthesis or secretion of 20-hydroxyecdysone (20E), JH, and other hormones. The many types of hormones in the insect body cooperate to ensure normal growth, development, reproduction, and other life processes in each insect.

Insect peptide hormones are a subset of neuropeptides, which are expressed and secreted by the central and peripheral nervous systems and then act on other organs, including the PG, corpus allatum, corpora cardiaca, and other glands. The insect gut is divided into three parts: the foregut, midgut, and hindgut. The gut is responsible for digesting food and absorbing nutrition; it is also regulated by neuropeptide hormones (as in mammals) (Audsley and Weaver, 2009; Nassel and Winther, 2010; Mayer, 2011; Pool and Scott, 2014), which is manifested in the peristalsis of the gut, cellular secretion of digestive enzymes, and intake of nutrition. In addition, JH and ecdysone affect the gut. For instance, JH can stimulate proliferation of enterocytes in the mated *D. melanogaster* females (Reiff et al., 2015); ecdysone can promote the midgut programmed cell death (Nicolson et al., 2015). The insect gut can also produce hormones, including peptide hormones, that ensure a normal physiological state, especially after intake of different foods. Furthermore, peptide hormones produced by the gut can act on other tissues, including nerve tissues; therefore, some peptide hormones produced by the gut are regarded as brain-gut peptides. There have been many studies and reviews regarding insect hormones. Peptide hormone genes and their receptors in insects have been reviewed previously (Riehle et al., 2002; Reiher et al., 2011; Wegener and Veenstra, 2015; Strand et al., 2016). Although there have been several reviews on hormones produced by the intestinal endocrine cells (Wegener and Veenstra, 2015), the most recent literatures has not yet been summarized. This review discusses recent research regarding peptide hormones related to the insect midgut. Specifically, this review describes the functions of peptide hormones produced in the insect gut, which provides a reference for future works on the molecular mechanisms of insect gut peptide hormones and the application for the pest biological control.

## Peptide Hormones Produced in the Gut

The insect digestive tract secretes many enzymes to digest proteins, lipids, and carbohydrates, which are digested extracellularly (Weidlich et al., 2013, 2015; Holtof et al., 2019). In the insect midgut, epithelial cells can produce several digestive enzymes and direct distribution of nutrients and transport of ions and water (Caccia et al., 2019).

Generally speaking, insect midgut cells include intestinal endocrine cells, intestinal epithelial cells, columnar cells with intestinal villi, and intestinal stem cells (Billingsley and Lehane, 1996; Caccia et al., 2019). Some insects can regulate intestinal pH via goblet cells (e.g., *Lepidoptera*) or copper cells; endocrine cells can produce peptide hormones (Huang et al., 2015; Caccia et al., 2019). In addition to the relationship between endocrine cells and hormones, columnar cells can secrete the signaling protein Hedgehog to regulate nutrient availability and developmental timing (Rodenfels et al., 2014); columnar cells also produce and secrete digestive enzymes, lysozyme, and antimicrobial peptides, and are able to absorb nutrition (Caccia et al., 2019).

The critical mechanisms of peptide hormones secreted by the insect gut have been relatively well studied in *Drosophila*. The gut endocrine cells of *Drosophila melanogaster* can produce six peptides, including allatostatins A, B, and C, neuropeptide F, diuretic hormone 31 (Dh31), and tachykinin (Veenstra et al., 2008). However, more than 45 neuropeptide genes were recently found in the genome of *D*. *melanogaster*. Based on reverse transcription polymerase chain reaction and *in situ* hybridization analyses, there are at least 10 neuropeptides expressed in midgut endocrine cells (Chen et al., 2016). Endocrine cells in different parts of the intestine produce distinct peptide hormones. For instance, Dh31, CCHamide-1 (CCHa1), Allatostatin A (AST-A), and Myoinhibiting peptide (MIP = Allatostatin B) are expressed in the posterior midgut; MIP is also expressed in middle midgut, and the whole midgut can produce AST-C, CCHamide-2 (CCHa2), and tachykinin (Chen et al., 2016). CCHa1 and CCHa2, as brain-gut peptides, are expressed in both midgut and in brain nerves (Ren et al., 2015). Use of the CRISPR/Cas9 gene editing system to disrupt CCHa1 and CCHa2 led to significant reduction in the food intake of CCHa2 mutants. Moreover, CCHa2 mutations can delay the development of larva, which may be related to the 80% reduction in mRNA concentrations of insulin-like peptides (ILPs) 2 and 3 induced by *ccha2* mutation (Ren et al., 2015). Therefore, peptide hormones secreted by the insect gut are very important for development and physiological functions. Table 1 provides a detailed introduction of several important peptide hormones produce by insect gut cells.

**TABLE 1:** Peptide hormones produced in midgut of insects.

**Peptide**	**Location**	**Function**	**Insect species and references**
Allatotropin	Digestive tract	Control JH biosynthesis	*Spodoptera frugiperda* (Abdel-Latief et al., 2004)
Allatostatin A, B(MIP), C	A: Endocrine cells in the posterior midgut	A: Regulate gut contraction, K^+^ absorption.	*Diploptera punctate*, *Aedes aegypti*, *Anopheles albimanus*, *Drosophila melanogaster* (Reichwald et al., 1994; Hernandez-Martinez et al., 2005; Spit et al., 2012; Vanderveken and O’Donnell, 2014; Nouzova et al., 2015)
	B: Endocrine cells in the midgut	B: Inhibit fore- and hind-gut contractions and food intake.	
	C: Entire midgut	C: Inhibit the synthesis of JH III	
CCHamide 1, 2	1: Posterior midgut	1. Regulate gut muscle contractions	*Drosophila melanogaster* (Reiher et al., 2011; Veenstra and Ida, 2014; Chen et al., 2016)
	2: Entire midgut	2. Digestion, release to hemolymph	
Neuropeptide F	Endocrine cells of the midgut	Release to hemolymph, modulate the physiology of feeding and digestion	*Helicoverpa zea* (Huang et al., 2011)
Orcokinin	Enteroendocrine cells, anterior and middle midgut	Regulate ecdysis	*Bombyx mori*, *Drosophila melanogaster*, *Rhodnius prolixus* (Yamanaka et al., 2011; Chen et al., 2015; Chen et al., 2016; Wulff et al., 2018)
Ryamide	Enteroendocrine cells of the anterior midgut	Regulation of feeding and digestion	*Bombyx mori* (Roller et al., 2016)
Tachykinin-related peptides	Midgut	Adaptation to different nutritional conditions; development	*Drosophila*, *Bombyx mori* (Van Loy et al., 2010; Nagai-Okatani et al., 2016; Yamagishi et al., 2018)
Diuretic hormone 31	Midgut endocrine cells	Regulate midgut contraction frequency	*Drosophila melanogaster* (Reiher et al., 2011; Vanderveken and O’Donnell, 2014)
Kinin	Hindgut	Regulate diuretic, digestive and myotropic activities and hindgut contractions	*Rhodnius prolixus* (Bhatt et al., 2014)
Myosuppressin	Posterior midgut	Regulate contractions of the anterior midgut and hindgut	*Rhodnius prolixus* (Lee et al., 2012)
Insulin-like peptides 2, 4, 5	Midgut	Related to lifespan, body size, growth	*Drosophila melanogaster* (Brogiolo et al., 2001; Zhang et al., 2009; Gronke et al., 2010)
Prothoracicotropic factors (PTTH)	Proctodaea/gut	Promote ecdysone and 3-dehydroecdysone production in the PG	*Ostrinia nubilalis*, *Lymantria dispar*, *Sesamia nonagrioides* (Gelman et al., 1991; Perez-Hedo et al., 2010)
Head peptide	Midgut endocrine cells	Inhibit host-seeking behavior	*Aedes aegypti* (Stracker et al., 2002)

**Most peptide hormones are also expressed in the nervous system, which is not marked in this table.**

### Insulin-Like Peptides

Insulin-like peptides (ILPs) are important peptide hormones in insects that play roles in metabolism, growth, reproduction, and aging (Garofalo, 2002; Defferrari et al., 2016; Nassel and Vanden Broeck, 2016; Zhang et al., 2017). ILP generation is regulated by insulin/insulin-like growth factor signaling (Nassel and Vanden Broeck, 2016). The insulin signaling pathway plays an important role in insect development and organ growth. Garofalo analyzed the genetics of insulin signaling in *Drosophila*, and compared the insulin signaling pathways among different species (Garofalo, 2002). Lin and Smagghe reviewed these pathways in detail (Lin and Smagghe, 2018).

ILPs can be expressed in the midgut and other tissues, where they can concurrently regulate the growth and development of insects. *D. melanogaster* has eight ILPs (DILP1–8) and two receptors, but mammals have many more receptors (Nassel and Vanden Broeck, 2016). The ILP1 gene of beet armyworm (*Spodoptera exigua*) can encode a 95-amino acid peptide, which is expressed in the fat body and epidermis, but not in the blood cells or intestine (Kim and Hong, 2015). When 5th-stage larvae of *S. exigua* were starved for 48 h, the transcription of SeILP1 was reduced, whereas the content of trehalose in hemolymph was increased more than twofold. Furthermore, RNAi of SeILP1 resulted in a significant increase in the content of trehalose in hemolymph. These findings showed that SeILP1 has an inhibitory effect on trehalose levels in hemolymph (Kim and Hong, 2015).

Factors released by the gut and adipocytes of *Drosophila* can regulate glucose-mediated DILP secretion. For example, DILP2 is produced by brain nerve cells, DILP3 is produced by gut muscles, DILP5 is produced by ovaries and Malpighian tubules, and DILP6 is mainly produced in the fat body (Nassel and Vanden Broeck, 2016). The expression levels of *dilp4* and *dilp5* in the gut of *Drosophila* larvae are high, whereas the expression level of *dilp6* in the gut is low; in the embryo stage, *dilp2* is highly expressed in the midgut (Brogiolo et al., 2001). Thus far, there have been multiple studies on DILPs produced by the brain and fat body, but few studies on DILPs produced by the intestine (Nassel and Vanden Broeck, 2016).

Although there are various mechanisms by which nutrients can regulate insect body size, the insulin/insulin-like growth factor signaling pathway and the target of rapamycin pathway both respond to food nutrition levels and regulate growth rate and duration; they also regulate the synthesis and concentrations of other important developmental hormones (Koyama and Mirth, 2018). The insulin receptor of *Drosophila* regulates body size and organ size by altering cell number and size (Brogiolo et al., 2001). In addition, ILP can regulate vitellogenin production and oviposition of the green lacewing, *Chrysopa septempunctata* (Zhang et al., 2017). In female *Drosophila*, DILP6 can regulate the metabolism of JH and dopamine (Rauschenbach et al., 2017). Mutation of *dilp6* reduced JH hydrolysis activity in mutant female *Drosophila*; it also increased dopamine synthesis enzymes activity. Under heat shock treatment, the reproductive capacity of females also increased. However, the fecundity and heat stress ability of the mutant flies did not differ from the characteristics of control flies when they were fed with precocene (JH inhibitor) for 10 h. Therefore, DILP6 can promote JH degradation and hinder its synthesis.

### Other Peptide Hormones

Nerves are distributed throughout the insect gut, and the central and gastrointestinal nervous systems can affect the insect gut. Peptide hormones control food intake and digestion; therefore, destroying specific nerves can reduce eating behavior (Spit et al., 2012). In addition to regulating the nervous system via the intestinal tract, the peptides produced by various parts of the intestinal tract function to regulate the digestive system itself. These peptides are typically expressed in precursor form and are activated when needed (Reiher et al., 2011). For example, in female black blowfly (*Phormia regina*) adults that have fed on beef liver, the midgut releases a peptide hormone with a relative molecular weight of 5,000–11,000 that acts on the brain and causes a neuroendocrine cascade reaction to promote egg formation (Yin et al., 1994). In the pre-molting and molting periods of the arthropod blue crab, *Carcinus maenas*, the level of hemolymph hyperglycemic hormone is more than 100 times higher than that in other developmental periods. Crustacean hyperglycemic hormone is released by endocrine cells in specific areas of the foregut and hindgut, which may be involved in molting (Chung et al., 1999). In the hindgut of European corn borer (*Ostrinia nubilalis*) and Gypsy moth (*Lymantria dispar*), a large number of prothoracicotropic factors exist; these prothoracicotropic factors can promote ecdysone and 3-dehydroecdysone production in the PG of the moth (Gelman et al., 1991). If the head of a gypsy moth is tied sufficiently to prevent brain-generated prothoracicotropic hormone from acting on the PG, the larva can also be induced to progress from molting to pupation when injected with intestinal extracts (Gelman et al., 1991). In summary, the gut can accept peptide hormones produced by other tissues to affect growth and development; similarly, the gut can produce peptide hormones that can regulate the physiological functions of insect development.

It was originally presumed that tachykinin-related peptide was produced by the midgut and released into hemolymph circulation to enable adaptation to different nutritional conditions (Winther and Nassel, 2001). The expression of tachykinin-related peptide in the midgut of silkworm has been demonstrated (Nagai-Okatani et al., 2016). When silkworm larvae midgut is incubated *in vitro* with nutritive substances such as glucose, amino acids, or the plant secondary metabolite chlorogenic acid, the midgut can discriminate and the endocrine cells secrete tachykinin-related peptide into the buffer of the cultured gut (Yamagishi et al., 2018). Prothoracicotropic hormone promotes the production of ecdysone by activating the target of rapamycin-receptor/ERK pathway to regulate metamorphosis (De Loof et al., 2015). The larvae and pupae of noctuid (*Sesamia nonagrioides*) can molt without the presence of the brain, although the PG is needed; this indicates that prothoracicotropic hormone can be produced in other tissues. Further analysis showed that prothoracicotropic hormone could be expressed in the intestine (Perez-Hedo et al., 2010).

With the sequencing of a growing number of insect genomes, 35 genes were found to encode regulatory peptides, among which five are ILPs (Riehle et al., 2002). The head peptide gene was identified in the genome of *Anopheles gambiae*; recombinant and purified head peptide was then injected into female mosquitoes that were not fed with blood, which prevented them from finding a host (Riehle et al., 2002). The head peptide gene of *Aedes aegypti* is expressed in the brain, ganglion end, and midgut (Stracker et al., 2002). This localization suggests that head peptide may be involved in more complex functional regulation; thus, additional meaningful peptide hormones may be identified by making full use of genomic information.

## Peptide Hormones Produced in Other Tissues and Affecting the Gut

During insect growth and development, some hormones can also affect intestinal activity. For example, ion transport peptide, a peptide hormone in *Drosophila*, is an antidiuretic peptide expressed in the brain, abdominal nerve cells, and other nervous areas. When *Drosophila* experiences thirst, ion transport peptide gene expression levels increase, regulating water balance by promoting water intake and inhibiting food intake and water excretion (Galikova et al., 2018). When ion transport peptide is overexpressed, water balance can be maintained by regulating defecation times. RNA interference to modify ion transport peptide levels can cause food to pass through the intestine quickly, similar to diarrhea (Galikova et al., 2018).

Interestingly, some social insects [e.g., ants (*Camponotus floridanus*)] can exchange certain hormones through oral fluid exchange (trophallaxis); the oral fluid contains various microRNAs and JH (LeBoeuf et al., 2016). The ingested RNA can be absorbed by the gut cells and then transferred to hemolymph (Maori et al., 2019). Compared to other social insects, many of the proteins in oral fluid are related to the regulation of growth, development, and maturation (LeBoeuf et al., 2016; He et al., 2018; Maori et al., 2019).

### Adipokinetic Hormone

Adipokinetic hormone (AKH) is a type of neuropeptide produced by the corpora cardiaca. It can mobilize energy metabolism in the fat body in many insects; it can also act on other tissues, such as the PG and aorta of fly larva, and the brain and crop of *Drosophila* adults (Lee and Park, 2004). AKH influences the intestine, mainly by controlling the activity of digestive enzymes in the intestine (Kodrik et al., 2012; Bodlakova et al., 2017). When Pyrap-AKH is injected into the firebug (*Pyrrhocoris apterus*), the amount of protein and lipids in the midgut increase; the level of triacylglycerols are mainly increased in the fore-midgut, while diacylglycerols are in the middle-midgut. AKH has no effect on lipase activity, but increases peptidase and glucoamylase activity in the middle and hind midgut (Kodrik et al., 2012).

Under starvation conditions, flies without AKH neurons do not show hyperactivity to search for food like wild-type flies, and they are resistant to starvation-induced death (Lee and Park, 2004). AKH is very important for maintaining basic nutrition levels; therefore, mutating the amino acid sequence of AKH in *Drosophila*, or constructing AKH-deficient and overexpressing *Drosophila* mutants, can promote the study of AKH mechanisms (Sajwan et al., 2015; Mochanova et al., 2018). For example, in blood-sucking insect (*Rhodnius prolixus*), the expression of AKH receptor *RhoprAkhr* in the fat body increases with triacylglycerol mobilization in the starvation state; if the AKH receptor gene is knocked down, triacylglycerol will accumulate in the fat body in the starvation state. However, if synthetic Rhopr-AKH is simultaneously injected into *R. prolixus*, the transcription levels of acyl-CoA-binding protein-1 and mitochondrial glycerol-3-phosphate acyltransferase, which are involved in fat metabolism, can be altered (Alves-Bezerra et al., 2016).

AKH can act on the intestine and influence digestive enzyme activity through AKH receptors present in the intestine. AKH receptor is a specific G protein-coupled receptor that exists on the target cell membrane. AKH receptors of the tsetse fly *Glossina morsitans morsitans* (i.e., *Glomo-akhr-a* and *Glomo-akhr-b*) are splicing variants of an open reading frame (Caers et al., 2016). AKH/corazonin-related peptide is the intermediate of AKH and corazonin, which is homologous to gonadotropin-releasing hormone of vertebrates. The AKH/corazonin-related peptide receptor gene of *A*. *aegypti* can form three variants through selective splicing of nine exons (Wahedi and Paluzzi, 2018). AKH/corazonin-related peptide and its receptor are mainly expressed in the central nervous system of *R. prolixus*; AKH/corazonin-related peptide is also expressed in the hindgut and posterior midgut (Zandawala et al., 2015). AKH can induce carbohydrate and fat metabolism only by binding to a rhodopsin-like G protein-coupled receptor (Marchal et al., 2018). In a study of AKH receptors of seven representative insect species, each receptor was able to be activated by endogenous AKH at very low doses. However, AKH receptors of mosquitoes could only be activated by endogenous ligands, while AKH receptors of locusts could be activated by AKHs of other insects (Marchal et al., 2018).

Hormones also play an important role in oxidative stress. AKH is the main stress hormone in the response pathway of oxidative stress (Kodrik et al., 2015); relevant work requires further study.

### Allatostatin

Although allatostatins were first found in the cockroach (*Diploptera punctata*) brain where they inhibit the production of JH by the corpus allatum, allatostatins are also expressed in midgut endocrine cells and other tissues (Reichwald et al., 1994). There are three types of allatostatins: A, B, and C (Hernandez-Martinez et al., 2005). The C-terminus of allatostatin type A (AST-A) is a conserved FGL amine motif, which can be detected in the brain and midgut of insects; its receptor is a G protein-coupled receptor. AST-A peptide, which can inhibit the secretion of JH, can also regulate food intake through inhibition or reduction of food intake (Felix et al., 2015). After *Anopheles* mosquitoes sucked blood, the expression levels of AST-A receptor genes *GPRALS1 and 2* in the midgut were significantly higher than after the mosquitoes ingested glucose, however, these expression levels were reduced in the head and ovary (Felix et al., 2015). Therefore, AST-A may be involved in the digestion of blood by the midgut.

### Other Hormones

In addition to the abovementioned hormones, several other hormones play important roles in regulating the physiological functions of insects. Both diuretic hormone 44 and leucokinin can induce the secretion of fluid from the Malpighian tubules; they can also regulate stress, feeding, rhythm, and other behaviors. Ganglion cells in the abdomen can produce both diuretic hormone 44 and leucokinin peptides simultaneously. Research has shown that knockdown of diuretic hormone 44 causes reduction of food intake, while knockdown of leucokinin does not; conversely, leucokinin knockdown can increase water retention (Zandawala et al., 2018). Thus, hormones produced by abdominal ganglion cells can affect the digestive system and homeostasis.

Ecdysone is synthesized by the PG; in addition, PG production of ecdysone is regulated by many neuropeptides. Neuropeptides mainly affect PG activity through hormones and neural pathways. For example, a group of neuropeptides identified in the silkworm, orcokinins, are neuronal prothoracicotropic factors that are mainly secreted by neuroendocrine cells in ventral ganglia; orcokinins can also be produced by small nerves of the central nervous system, as well as by endocrine cells of the midgut (Yamanaka et al., 2011). Orcokinins can regulate ecdysis in the hemimetabolous insect, *R. prolixus*, through the peptidergic signaling pathway (Wulff et al., 2017). Orcokinin neuropeptides are very important in the molting activity of *R. prolixus*. The *Orcokinin* gene can form two different neuropeptide precursors through selective splicing. RhoprOKA affects differential expression of molting-related neuropeptide precursors. RhoprOKB is related to feeding and midgut physiological function, since OKB is a brain-gut peptide in insects that can improve the frequency of spontaneous peristalsis and contraction of the midgut (Wulff et al., 2018). Endocrine cells with orcokinin B/C-like immune activity in the fore-midgut were identified by immunohistochemistry. The number of immune-active cells after 1 h of blood sucking was lower than that without feeding, which may be due to the release of OKB peptide from cells (Wulff et al., 2018).

## Hormones Produced by Intestinal Symbionts

In the long evolutionary process, there have been many symbionts in the insect gut. Symbionts inhabiting the gut can help insects digest food, regulate growth and development, prolong life, detoxify, immunize, and communicate (Engel and Moran, 2013; Wu et al., 2016). Insects provide nutrition and habitats for symbiotic bacteria; these bacteria also affect their hosts. The gut symbionts of insects are regulated by molecules in midgut epithelial cells, the most important of which are reactive oxygen species and immune deficiency pathway-produced antimicrobial peptides (Huang et al., 2015). The existence of intestinal bacteria can affect the development of the midgut; for example, these bacteria can induce the proliferation of intestinal stem cells and the renewal of epidermal cells. In honey bee (*Apis mellifera*), a γ-proteobacterial species can encode pectin-degrading enzymes, which may be related to the degradation of pollen walls to help pollinate crops (Engel et al., 2012).

Rational use of honeybee symbiotic bacteria may help to improve the health conditions of honeybees with respect to various factors, including pathogens, parasites, pesticides, environmental changes, and habitat loss (Crotti et al., 2013). In addition, the intestinal microorganisms of the honeybee can promote individual weight gain by mediating changes in vitellogenin production, insulin signaling, and the gustatory response of the host (Zheng et al., 2017). Compared with sterile bees, metabonomic analysis revealed that the short-chain fatty acids produced by the intestinal bacteria of normal bees affect the gut environment (e.g., by reducing intestinal pH and redox capacity). This environmental change alters the insulin/insulin-like growth factor signaling pathway. Honeybees have two ILP genes and two putative insulin receptors. In addition, vitellogenin production is closely related to the insulin/insulin-like growth factor signaling pathway. The expression levels of *ilp1* and *Vg* in the head and abdomen were shown to be several times higher in normal bees than in sterile bees; the expression levels of *ilp2* and *inR1* were also higher in normal bees (Zheng et al., 2017).

In addition to the above-described metabolite regulation, symbiotic bacteria can affect the growth and development of the host through the use of bacterial enzymes. In *Drosophila*, the symbiotic bacterium *Acetobacter pomorum* can regulate insulin/insulin-like growth factor signaling, body size, energy metabolism, and intestinal stem cell activity through PQQ-ADH enzyme activity (Shin et al., 2011).

Gut microbes can stimulate gut nerves to transmit signals to brain nerves (Forsythe and Kunze, 2013). In mammals, the vagus nerve mediates the connection between intestinal bacteria and the central nervous system. However, in insects, gut bacteria exhibit clear regulation of host behavior. Some pheromones produced by symbiotic bacteria in the gut can affect insect behavior. For example, *Pantoea agglomerans*, a bacterium isolated from the gut of locusts, produce a mixture of cohesion pheromone (guaiacol, which causes locusts to gather) and a small amount of phenol when added to sterile feces; however, the mixture cannot be produced by addition of bacteria pathogenic to locusts (Dillon et al., 2002). In *Drosophila*, through ingestion of different foods, biases in adult mating can be induced. For example, *Drosophila* fed sucrose medium tend to mate with *Drosophila* fed the same medium, while *Drosophila* fed starch medium tend to mate with others fed starch medium. However, addition of antibiotics to food can inhibit this phenomenon, which may be related to changes in sex pheromone levels from symbiotic bacteria, such as *Lactobacillus plantarum* (Sharon et al., 2011).

## Hormones and Immunity

Insects rely on innate immunity to resist the invasion of pathogens, but the immune level of an individual is closely related to development. For the silkworm, the immune level of the midgut changes dynamically in the stages of larva molting, mulberry eating, and wandering, in accordance with the expression of antimicrobial peptide (Xu et al., 2012; Yang et al., 2016). Injection of ecdysone into newly molted silkworm larvae can lead to induction of the larvae-to-larvae molting stage; gene expression differences in the midgut are also similar (Yang et al., 2016). In the prophase of molting, c-type lysozyme and pyrrhocoricin-like antimicrobial peptide are significantly increased in the midgut, which could reduce the number of *Burkholderia* intestinal symbionts (Kim et al., 2014). Because injection of 20-hydroxyecdysone can reduce the number of intestinal bacteria (Kim et al., 2014), changes in immune levels are presumably related to hormone changes. In addition to the gut, changes in hormone levels appear to enhance the cellular immunity of insects. When ecdysone is added to the *Drosophila* l(2)mbn cell line, the phagocytosis ability and antimicrobial peptide expression can be enhanced under immune stimulation (Dimarcq et al., 1997).

However, during the process of insect reproduction, hormone regulation reduces immune levels (Schwenke and Lazzaro, 2017). For example, during reproduction in female *Drosophila*, mating, semen protein, and sex peptide lead to reduced systemic immune activity against bacteria (Short et al., 2012; Schwenke et al., 2016). Sex peptide can stimulate the synthesis of JH in female flies after mating. However, JH also inhibits immunity because JH can reduce the humoral immunity and especially antagonize the 20E-dependent immune activities (Rolff and Siva-Jothy, 2002; Flatt et al., 2008). Therefore, after mating, female flies are more likely to be infected with pathogens and die (Fedorka et al., 2007; Miest and Bloch-Qazi, 2008; Schwenke and Lazzaro, 2017). If JH signaling is blocked, the survival rate of female flies that have engaged in mating will recover after pathogen infection (Schwenke and Lazzaro, 2017). The seminal fluid of male *Drosophila* contains sex peptide, which can be transferred to female flies during mating. Sex peptide can bind to specific sites of the central nervous system, peripheral nervous system, and reproductive tract of females. Receptors in the nervous system can recognize and activate the signal pathway after mating. In addition, receptor proteins in the genital tract can transfer sex peptide to hemolymph (Kubli, 2003).

## Peptide Hormones and Pest Control

The harm from abuse of traditional chemical insecticides is becoming more and more serious, and new biological insecticides have been given increasing attention due to their green and friendly characteristics. Studies have increasingly shown that ecdysone, JH, and their analogs can be used for biological control of pests (Dhadialla et al., 1998; Retnakaran et al., 2003; Wilson, 2004). With respect to hormone regulation of insect reproduction, energy metabolism, water balance, feeding behavior, sexual attraction, and growth and development, Gade and Goldsworthy proposed in 2003 that insect peptide hormones could be used to design new, safe, and selective complexes for insect pest control (Gade and Goldsworthy, 2003). A normal level of allatostatin is critical for the growth and development of insects. Mannose-binding lectin (*Galanthus nivalis* agglutinin) can bind to the intestinal epithelial cells of insects and then enter hemolymph. Feeding the purified fusion protein of *G. nivalis* agglutinin and allatostatin to *Lacanobia oleracea* larvae can inhibit their feeding and growth, however, there is no such effect when *G. nivalis* agglutinin or allatostatin are fed alone, or when they are fed together (Fitches et al., 2002).

Kinin neuropeptide is a type of neuropeptide with a Phe^1^-Xaa^2^-Yaa^3^-Trp^4^-Gly^5^-NH_2_ structure at the C-terminus, which exists in many insects (Zhang et al., 2015a). It can promote the excretion of urine and stimulate contraction of the hindgut, as well as release of digestive enzymes. Zhang et al. designed and synthesized insect kinin analogs via a peptidomimetic strategy and obtained a biological insecticide with high aphid-killing activity: analog II-1. Its LC_50_ is 0.019 mmol/L, which is lower than that of commercial pymetrozine (LC_50_ = 0.034 mmol/L) (Zhang et al., 2015a).

When AKH and the pathogenic nematode *Steinernema carpocapsae* were applied to adults of *P. apterus*, the mortality rate of the adults increased 2.5-fold within 24 h; in addition, the metabolism of the insects was intensified and the production of carbon dioxide increased in the insect body. However, if AKH receptor expression is knocked down in *P. apterus*, the mortality rate is significantly reduced (Ibrahim et al., 2017).

## Conclusion

Peptide hormones have been shown to cause no chemical pollution and to have specific action. Therefore, hormonal peptides can be used as new and safe biological insecticides; relevant research requires greater attention. The production and functions of peptide hormones are becoming increasingly clear, especially with respect to the mechanisms of action of steroids and several neuropeptides. In addition to the functions discussed above, insects have certain peptide-regulated behaviors; for example, insect SIFamide is a neuropeptide produced by four medial interneurons, which can affect sexual behavior, sleep, death, and pupa number (Lismont et al., 2018). Insect diapause hormone is a type of peptide that can help individuals survive in a changing environment, such as overwintering; some insects exhibit embryonic diapause, while others exhibit pupal diapause (Zhang et al., 2015b). The research regarding mechanisms underlying these behaviors in insects is very important for understanding human life activities, as well as for biological control.

Some neuropeptides and peptide hormones of insects can be used to treat mammalian diseases, as they exhibit antifungal, antitumor, and antiviral properties (Chowanski et al., 2016). Notably, in addition to peptide hormones, insects can produce antimicrobial peptides. Insects have no adaptive immunity to produce antibodies, however, they can produce antimicrobial peptides through the innate immune system, which eliminate pathogens from the body. Insect antimicrobial peptides also reportedly have bactericidal effects on many human pathogenic bacteria (Chowanski et al., 2016). Some antimicrobial peptides, such as CopA3, were found to increase the proliferation of colonic epithelial cells and enhance intestinal mucosal barrier function (Kim et al., 2016). CopA3 of Dung beetle (*Copris tripartitus*) is a 9-mer disulfide dimer peptide. *In vitro*-synthesized CopA3 can increase the proliferation of HT29 cells, as well as the levels of proliferation and apoptosis of colonic epithelial cells in mice treated with CopA3; these findings suggest that CopA3 can promote the regeneration of mouse colonocytes, given that CopA3 can downregulate the cyclin-dependent kinase inhibitor p21^Cip1/Waf1^. Moreover, CopA3 could prevent enteritis in mice that were treated with *Clostridium difficile* toxin A (Kim et al., 2016).

The study of insect peptide hormones is also very useful in analysis of decapods. Neuropeptides exhibited significant similarity among seven shrimp and crab species; these included elongated pigment dispersing hormone, a newly identified peptide. Compared with insects, the only neuropeptides not found in decapods were allatotropins (Veenstra, 2016).

At present, research on insect hormones is mainly focused on the interactions of JH, ecdysone, and other hormones, while studies on peptide hormones in the midgut have been sparse and unsystematic. However, midgut hormones are important in nutrition regulation, water balance, development, and pest control. Therefore, research regarding midgut peptide hormones requires greater attention.

## Author Contributions

KW, EL, QL, SL, JW, YN, and WH wrote the manuscript.

## Conflict of Interest

The authors declare that the research was conducted in the absence of any commercial or financial relationships that could be construed as a potential conflict of interest.
